# Mechanisms of young professional engagement in health policy development: a cultural domain approach

**DOI:** 10.3389/fpubh.2024.1389649

**Published:** 2024-10-23

**Authors:** Yulia A. Levites Strekalova, Lina Modjarrad, Sara Midence

**Affiliations:** Department of Health Services Research, Management, and Policy, College of Public Health and Health Professions, University of Florida, Gainesville, FL, United States

**Keywords:** cultural domain analysis, engagement science, health policy development, emerging leadership, public health advocacy

## Abstract

**Introduction:**

Engagement in public health policy development is critical to forward-thinking public health policy. There is a robust empirical case to support the prospect of the engagement of young adults in civic and research efforts. However, the literature is limited in conceptualizing the mechanisms of engagement in health policy development from the perspective of young adults. This study applied the concepts and methods of cognitive anthropology to identify the mechanisms of engagement in health policy development from the perspective of young people.

**Methods:**

Online elicitation and cognitive domain analysis were applied to collect and analyze the data. Students enrolled in a public health leadership class at a large United States southeastern university were invited to participate in an online discussion. Saturation was achieved after the eighth submission. Data were analyzed qualitatively for repetition and recurrence and quantitatively to assess their cultural saliency.

**Results:**

Thirty-two students submitted 147 individual engagement ideas. The analysis resulted in 24 unique mechanisms organized into 10 clusters. The most prominent engagement mechanisms included education, peer activities, advocacy, direct involvement, and activism.

**Discussion:**

In the dynamic landscape of public health, active involvement in health policy development presents a crucial pathway for leadership among young professionals. The application of cognitive anthropology methods contributes to the emerging science of engagement and allows to identify and measure consensus around the pathways for engagement in health policy development among young professionals.

## Introduction

The state of the United States healthcare system reveals asymmetries between the nation’s most and least autonomous populations, partly due to systemic disparities. Among these upstream determinants is a historic underrepresentation of vulnerable groups, such as racial and ethnic minorities, individuals who grow up in economically impoverished and medically underserved areas, for whom health policy development would likely yield *greater (future) marginal benefit* than their more autonomous counterparts. Conversely, these vulnerable groups are made to shoulder diminished autonomy and disenfranchisement in the very political/social systems meant to serve them. Students and young professionals, for example, are critical stakeholders in the future of public health. Still, their political input has not been commensurate with the outputs of policies that directly or indirectly affect them. Particularly worth noting is the vulnerability of subgroups, including socioeconomically disadvantaged youth, both in the United States and in low-and middle-income countries (1). This literature review synthesizes the best available empirical research to support democratizing the political and scientific process to include younger populations.

Engagement in public health policy development is critical to forward-thinking public health policy, particularly as an antecedent to health equity. This need for engagement extends to young professionals whose relative inexperience should uphold the modern sensibility they bring to the policymaking process. Early-career professionals present a unique advantage of identifying with younger demographics more than an antiquated political process (2). One study surveyed 30 cross-national early-career pharmacists and pharmaceutical scientist groups (ECPGs), and several had policymaking roles in addressing regional and global health challenges (3). Proving scaffolding for policy engagement, academic institutions effectively offer programming for emerging scholars who can collaborate across professions on scientific and social policy (2). These findings speak to the merits of a younger workforce in developing contemporary health policy through their lived experiences, knowledge base, and awareness of priority health challenges (2). As such, youth and young adults represent a historically untapped voice in the development of health legislation and the implementation of subsequent quality and equity initiatives.

The need for the systematic study of the engagement mechanisms has been articulated before. Social-scientific studies that investigate the processes of research engagement, citizen science, and community-based participatory research provide a robust body of literature and engagement frameworks for the study of health policy development (1, 4, 5). Public health research is a conduit for civic engagement, which is necessary for developing well-informed policy that meets priority populations’ most imminent needs and concerns (5). Youth and young adults comprise a priority population and should, in turn, be better reflected in the scientific process that precedes or informs policy. Mandoh and colleagues describe Youth Advisory Groups (YAGs) as an adolescent-led strategy for designing, implementing, and interpreting research that boasts meaningful relevance to their age demographic (6). In other words, youth and young adults champion efforts targeted toward their health needs in an engaged, self-actualizing way (6). Such community advisory groups enable youth to spearhead research questions pertinent to their health needs, develop a positive self-concept, and promote youth leadership and a sense of empowerment.

Citizen science provides a different insight into the mechanisms of young adult engagement with policy development. Citizen science can be defined as a broad scope of activities that integrate the local or general public in the academic process (4). Mechanisms for citizen science are 3-fold and include: (1) consultation of public opinion; (2) collaboration through the democratization of power between scientists and the community; and (3) control, whereby citizens have a greater compelling voice in making administrative decisions that are of relevance to them (4). Consequently, community mobilization in the way of citizen science is beneficial, but not without its own ethical considerations (4). Achieving symmetrical power and giving voice to participating citizens is critical for the effectiveness of citizen science approaches (4). Another crucial component of ethical considerations is cultural competence, which has been well articulated within community-based participatory research (CBPR) (5). CBPR is distinguished by its emphasis on building community rapport and relational ties between academics and citizens (5). This approach diverges from antiquated field studies, where a “helicopter model” would historically involve academics conducting their research and leaving after that with nominal community involvement or benefit (5). Both citizen science and CBPR present a strong case for the need to empower citizens to identify priority health and policy needs and have a part in addressing them, whether proximally through groundwork or distally through policy change (7).

There is a robust empirical case to support the prospect of youth and young professional civic and research engagement on their health and personal agency. The engagement of young people in health policy development is of particular importance as they represent both the emerging leaders in policy development and future health policy beneficiaries. Literature supports that younger populations belong inside–not on the fringes–of the policy arena itself, and empowering younger populations to have a voice in health policy might prove pivotal in promoting health equity and slowing the burden of chronic diseases that young adults are shouldering at increasing rates (8). Furthermore, youth-policymaker partnerships can support the co-design of policies and legislation and become a feasible and practical roadmap if supported by equitable engagement mechanisms (9).

Given the current scope of literature, a multilevel and multidisciplinary approach to improving public health policy–vis-a-vis patient, civic, and youth engagement–has a robust evidential basis for success. However, there is a limited evidential basis exploring the mechanisms of youth engagement and experiences in policy development. This study stands to begin addressing this need and answer the following research question:

What are the mechanisms of young adult engagement in health policy development as viewed by young public health professionals?

## Methods

### Conceptual framework

Cultural domain analysis studies demonstrate how people in a cultural group think about things related to their society. Conceptually, the cognitive theory of culture posits that when multiple respondents are questioned independently, the recurrence and salience of their responses identify the group’s shared knowledge and constitute the group’s cultural domain, or “truth” (10). The theory and methods of cultural domain and consensus analysis evolved from the efforts of anthropologists to classify relationship structures in different cultures (11). This interest in organizing kinship systems led to methods for discovering sets of terms in other domains (12, 13). Cultural domain elicitation and cultural consensus analysis are integrated mixed methods that support the systematic assessment of the salience of specified cultural domains and shared knowledge. It is used to design and test connections within a cultural domain based on qualitative data collection and subsequent statistical analyses. Cultural consensus analysis can be utilized at different points in the program planning, implementation, and evaluation cycle to maximize proposed interventions’ local suitability, acceptability, and quality. Methodologically, the analysis provides aggregate estimates of the latent cultural knowledge at the group level while accounting for heterogeneity in informant competence. It has been used in various fields, such as cultural anthropology, social networks, linguistics, sociology, and psychology (14, 15).

### Participants and data collection

Students enrolled in a public health leadership class at a large United States southeastern university were invited to participate in an online discussion. Data collection for the present study followed the key methodological guidelines of cultural domain elicitation: (1) respondents were asked a question of an equal level of difficulty, and (2) informants answered the question individually (16). Students responded to the prompt: “Think of as many as possible ways for young adults and emerging public health professionals to get engaged in health policy development.” Students had to post a response before getting access to the responses of others. This process was designed to elicit ideas around policy engagement and allow the participants to post their opinions independently and asynchronously online. In line with the methodological recommendations for cultural domain elicitation, participants were asked to post responses individually, and reactions from others became visible once they submitted their responses. Participants were given 24 h to post their responses. After this time, comments were extracted into a Word document. Each comment was assigned a participant ID to maintain the anonymity of the respondents in the final dataset. Superfluous words were removed, and comments were broken apart into meaningful idea units and organized using a table listed participant ID, comment sequence, and idea (e.g., 01_1_[idea], 02_2_[idea]). Retaining the sequence in which the participants submitted the ideas was essential for the subsequent cultural domain and saliency analyses.

### Data analysis

Collected anonymous statements were read by two reviewers to document repetition (use of the exact words) and recurrence (use of different words that communicate the same idea) (17). When recurring statements were identified, they were consolidated into a single statement and organized in the order in which the participants submitted them. Next, Free List Analysis under R Environment Using Shiny (FLARES) software was used to analyze the statements and assess the saliency of the mechanisms of engagement in health policy development (18). FLARES is an open-source, cloud-based software that facilitates the identification of knowledge domains through systematic normalization of elicited cultural knowledge and subsequent quantitative analyses of idea salience. To prepare the data for the analysis, each response was assigned a unique ID, and ideas listed by each respondent were organized into lists maintaining the order in which they were mentioned. The data was reviewed repeatedly for the consistency of wording and normalized for capitalization and punctuation. Once the date was processed, FLARES was used to conduct the frequency of mention, saliency, and multi-dimensional scaling analyses.

### Positionality

The first author is a faculty member with a history of engaging undergraduate and graduate students in health-related research, program development, and evaluation efforts. This experience gives the first author the participant-observer viewpoint, which allows for the emersion and crystallization of practices into conceptual constructs. The second and third authors are graduate students whose positions align closely with the participants’ vantage points, thus supporting the diversity of perspectives considered in the present research.

### Ethical review

The protocol for this study was submitted for review by the Institutional Review Board of the University of Florida and received an exempt designation (#ET00022928). Data were obtained by retrospective record review, and all participant identifiers were removed prior to data analyses.

## Results

The dataset included 32 respondents for a total of 147 items with an average item length of 17 words (*M* = 17.69, *SD* = 15.45). Respondent lists had an average size of 4.6 (*SD* = 1.68). The lists included 24 unique items, as shown in Table 1. Eight respondents cited all 24 unique items, and the data from the remaining 24 respondents added no new information. The frequency of items ranged from 1 to 22 and the relative citation frequency ranged from 0.031 to 0.688. The top five most frequently mentioned items were “Become a volunteer or member of a community advocacy organization,” “Become a public health policy intern,” “Attend classes on health policy development,” “Connect with and write to public health officials,” and “Attend conferences and workshops with health policy professionals speaking.” Together, the 24 statements presented in Table 1 constitute a cultural domain instrument that represents the shared knowledge among young public health professionals around the salient mechanisms of engagement in policy development.

**Table 1:** Cultural domain instrument and saliency of items for mechanisms of engagement in health policy development.

Item. nr	Cited items	Citation frequency	Relative citation frequency	Mean rank	Smith’s index	B score
1	Advocate for health policy education	1	0.031	9	0.0063	0.0018
2	Attend classes on health policy development	13	0.406	3.154	0.2587	0.3053
3	Attend conferences and workshops with health policy professionals speaking	10	0.312	3.3	0.1868	0.2189
4	Attend local and county government meetings	2	0.062	1	0.0625	0.0476
5	Become a public health policy intern	17	0.531	2.882	0.3379	0.4001
6	Become a volunteer or member of a community advocacy organization	22	0.688	2.545	0.49	0.5542
7	Become informed in policies of interest	4	0.125	3.25	0.074	0.0794
8	Conduct policy research projects	8	0.25	4.625	0.1167	0.1547
9	Connect with and write to public health officials	13	0.406	3.385	0.2266	0.2832
10	Create educational materials explaining policies	5	0.156	4.2	0.069	0.086
11	Disseminate educational materials explaining policies	6	0.188	5	0.0761	0.1063
12	Follow local state and federal health policy news	7	0.219	3.857	0.1219	0.1468
13	Join a professional association or organization	7	0.219	1.571	0.1966	0.1909
14	Organize symposia and events	2	0.062	1	0.0625	0.0476
15	Participate in debates or town halls to practice discussing opinions	5	0.156	4.2	0.083	0.093
16	Participate in elections and voting	2	0.062	4	0.0302	0.0278
17	Participate in student and other peer organizations engaged in policy advocacy	6	0.188	3.333	0.1326	0.1424
18	Recognize policy effects on personal life	4	0.125	2.75	0.0688	0.0635
19	Seek employment in a health policy-oriented job	1	0.031	4	0.0078	0
20	Seek out mentors who are experienced in health policy	2	0.062	3	0.0292	0.0238
21	Shadow a public health professional	2	0.062	1	0.0625	0.0476
22	Support younger people getting elected to office	1	0.031	4	0.0125	0.004
23	Write about policy issues on social media	4	0.125	3.5	0.0547	0.0648
24	Write commentary and opinions on policy issues	3	0.094	6	0.0312	0.0426

**Figure 1:**
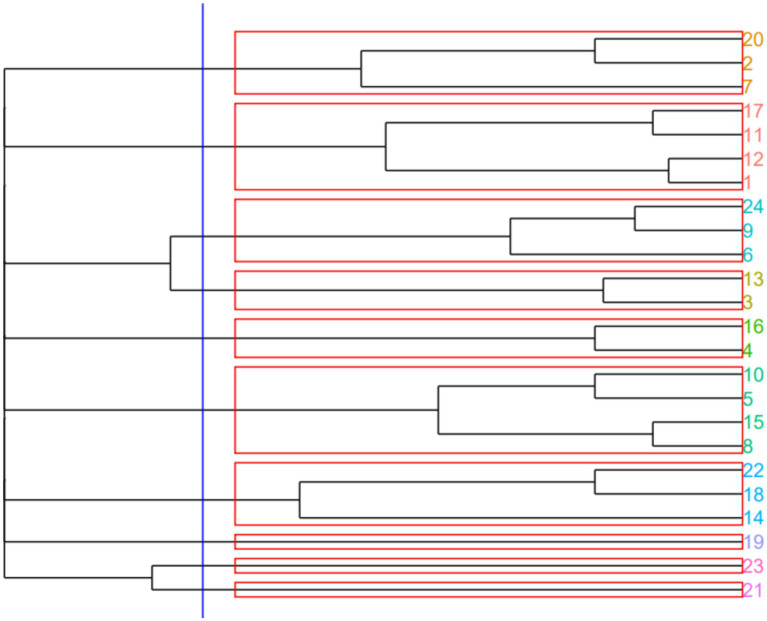
Dendrogram of item-by-item proximity using the Henley frequency of citation index. Policy engagement statements are organized into clusters. Refer to Table 1 for the corresponding IDs and full formulations of statements describing the mechanisms of engagement in health policy development.

Item-by-item proximity analysis resulted in 10 clusters tested for min *k* = 3 and max *k* = 10. The cluster dendrogram is shown in Figure 1, which presents the policy engagement statements organized into clusters (refer to Table 1 for the corresponding IDs and full formulations of statements describing the mechanisms of engagement in health policy development). Five clusters included three or more engagement mechanisms, which signals the opportunity to combine these strategies under one program. Based on the included mechanisms, the clusters were conceptualized as follows. Cluster 1 identified *education* as an essential mechanism for engagement in policy development through mentoring (statement 20), coursework (statement 2), and information consumption (statement 7). Cluster 2 identified *peer activities* as another engagement mechanism through student organizations (statement 17), dissemination of materials (statement 11), policy news consumption (statement 12), and advocacy for policy education (statement 1). Cluster 3 identified *advocacy* as another mechanism that involves organization membership (statement 6), outreach to public health officials (statement 9), and volunteering (statement 24). Cluster 4 included *direct involvement* as a mechanism for policy engagement through internships (statement 5), development of educational materials (statement 10), debates (statement 15), and policy research (statement 8). Finally, cluster 5 was conceptualized as *activism* as a mechanism that involves event organization (statement 14), application of policy to the personal sphere (statement 18), and support for young professionals as elected officials (statement 22).

## Discussion

This study explored the mechanisms of policy engagement used by young professionals to shape the future of public health. Applying the cognitive theory of culture to identify the shared perceptions of young public health professionals toward meaningful mechanisms of engagement in policy development, this study showed that the set of mechanisms for policy engagement is diverse. Yet, this study also suggests a high level of consensus among young professionals about the most salient strategies for engagement. In the dynamic landscape of public health, active engagement in health policy development presents a crucial pathway for leadership among young professionals. Participating in policy formulation gives young professionals practical experience in decision-making, critical thinking, and effective communication (8). This proactive role facilitates personal and professional growth. It empowers individuals to advocate for positive change and address pressing health issues on a broader scale, thus serving as a catalyst for cultivating essential leadership skills (3).

This research contributes to the growing body of literature that focuses on the conceptual and operational definition of engagement within the public health and health policy domain (19, 20). This study offered a novel perspective on engagement as a cultural phenomenon that can be systematically defined and measured as shared knowledge with the application of the cognitive theory of culture. Specifically, the evidence presented in this study shows that the mechanisms of engagement in public health policy development can be effectively elicited and analyzed using established social-scientific methods. The method and results presented in this study have implications for the growing science of engagement and applications for the practice of public health education and workforce development programs.

This study has conceptual, methodological, and practical implications. Conceptually, this research contributes to the broader body of literature focusing on the field of engagement. The clusters of engagement mechanisms presented in this study suggest a set of propositions that can be empirically tested in future studies. Specifically, future research can assess the relative contributions of education, peer engagement, advocacy, hands-on experiences, and activism toward sustained engagement of emerging public health professionals in policy development. Furthermore, the application of the cultural perspective represents a promising approach and an opportunity to systematically capture the endogenous perspectives of groups whose voices are underrepresented in policy development efforts. Specifically, cultural domain analysis considers young professionals’ unique perspectives and experiences, allowing for a more comprehensive understanding of their engagement in policy initiatives. This conceptual framework recognizes the value of collective insights and offers a more nuanced and effective means of measuring engagement in policy-related contexts.

Methodologically, this study provides an example of the application of cognitive anthropological methods to the development of a policy engagement instrument by directly involving the target respondent group (11). Policy engagement programs and broader citizen engagement initiatives frequently face challenges when identifying measures that accurately capture the specifics of their context and provide a meaningful representation of relevant metrics that reflect that context (21). This predicament becomes particularly pronounced in the realm of policy engagement involving young professionals. It becomes imperative to articulate meaningful pathways for engagement that resonate with the perspectives of these young professionals, as they bring a unique set of knowledge and experiences that may need to be fully leveraged or addressed by established professionals and academics who typically develop existing measures. Traditional approaches to measuring policy development are antiquated by a failure to reflect the emerging knowledge and experiences of today’s young professionals, thus undermining their validity. This study contributes to the methodological framework for cultural domain assessment and provides a practical approach to eliciting and discerning salient cultural knowledge for a precisely defined population.

The saturation for this study was reached after the inclusion of the first eight responses. This evidence highlights the effectiveness of the proposed methodological approach and underscores the efficiency with which this goal can be accomplished. Moreover, this study introduces a robust initial instrument that can be utilized to measure cultural consensus and the mechanisms of engagement in public policy development. This instrument serves as a solid foundation for future studies, which can then proceed to evaluate the consensus surrounding these items in a comprehensive and systematic manner.

Practically, this study has implications for the design and evaluation of policy engagement programs (22). Efforts directed toward engaging participants in policy studies can effectively utilize the mechanisms of engagement instruments to evaluate and assess the interests and efficacy of participants to pursue further opportunities in policy development, thereby ensuring their readiness and competence in this field. The instrument can also be applied to assess program logic models and potential programming gaps. By adopting cultural consensus analysis, policy engagement programs and broader citizen engagement initiatives can tap into young professionals’ generational shared knowledge and experiences. The cultural consensus methodology provides a platform for the synthesis and analysis of individual perspectives. In doing so, policy engagement programs can develop measures that resonate more effectively with young professionals and provide them with meaningful pathways for engagement. This analytical approach allows for a more inclusive and representative measurement framework, essential for fostering meaningful and impactful engagement among young professionals.

It is also worth mentioning this study’s conceptual and methodological limitations. First, this study collected data from respondents who attended one academic institution and degree program. A variety of the elicited engagement mechanisms suggests a diversity of perspectives among the respondents. However, a high level of consensus is also expected among young public health professionals whose professional development has significant overlaps. This similarity of experiences is aligned with the theoretical foundations of the cognitive theory of culture. However, future validation of the developed cultural domain instrument should include respondent groups with diverse backgrounds. The cultural domain instrument presented in this study constitutes the foundational, initial step in eliciting and operationalizing the mechanisms of engagement. However, future validation studies are necessary to assess the broad application of this instrument in a cultural domain.

In conclusion, the challenges faced by policy engagement programs and broader citizen engagement initiatives in accurately capturing the specifics of their context and measuring relevant metrics can be particularly pronounced when involving young professionals. Traditional approaches to policy development may need to pay more attention to the valuable insights and experiences that young professionals bring. Cultural consensus analysis offers a promising approach by adopting an endogenous perspective that leverages the shared experiences of young professionals. This method recognizes and values their collective insights, enabling the development of more nuanced and effective measures of engagement in policy-related contexts. By embracing cultural consensus analysis, policy engagement programs can foster meaningful pathways for engagement that resonate with the perspectives of young professionals, ultimately leading to more impactful and inclusive policy outcomes.

## Data Availability

The original contributions presented in the study are included in the article/supplementary material, further inquiries can be directed to the corresponding author.
